# Supplementary figures and images for: 3D reconstruction identifies loci linked to variation in angle of individual sorghum leaves
Source: PeerJ. 2021 Dec 22;9:e12628. doi: 10.7717/peerj.12628 (PMC8710048; doi:10.7717/peerj.12628)

(A)

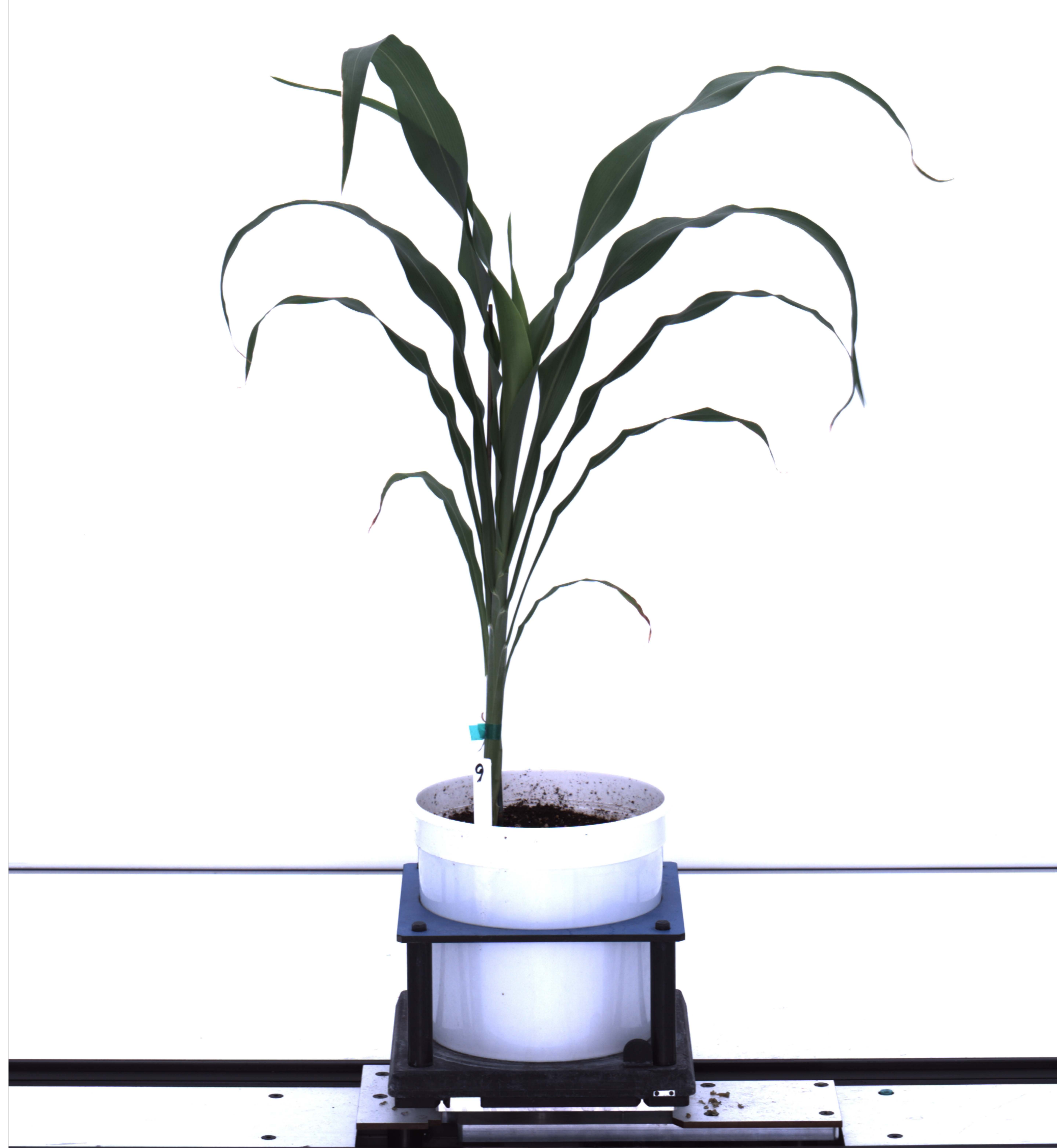

(B)

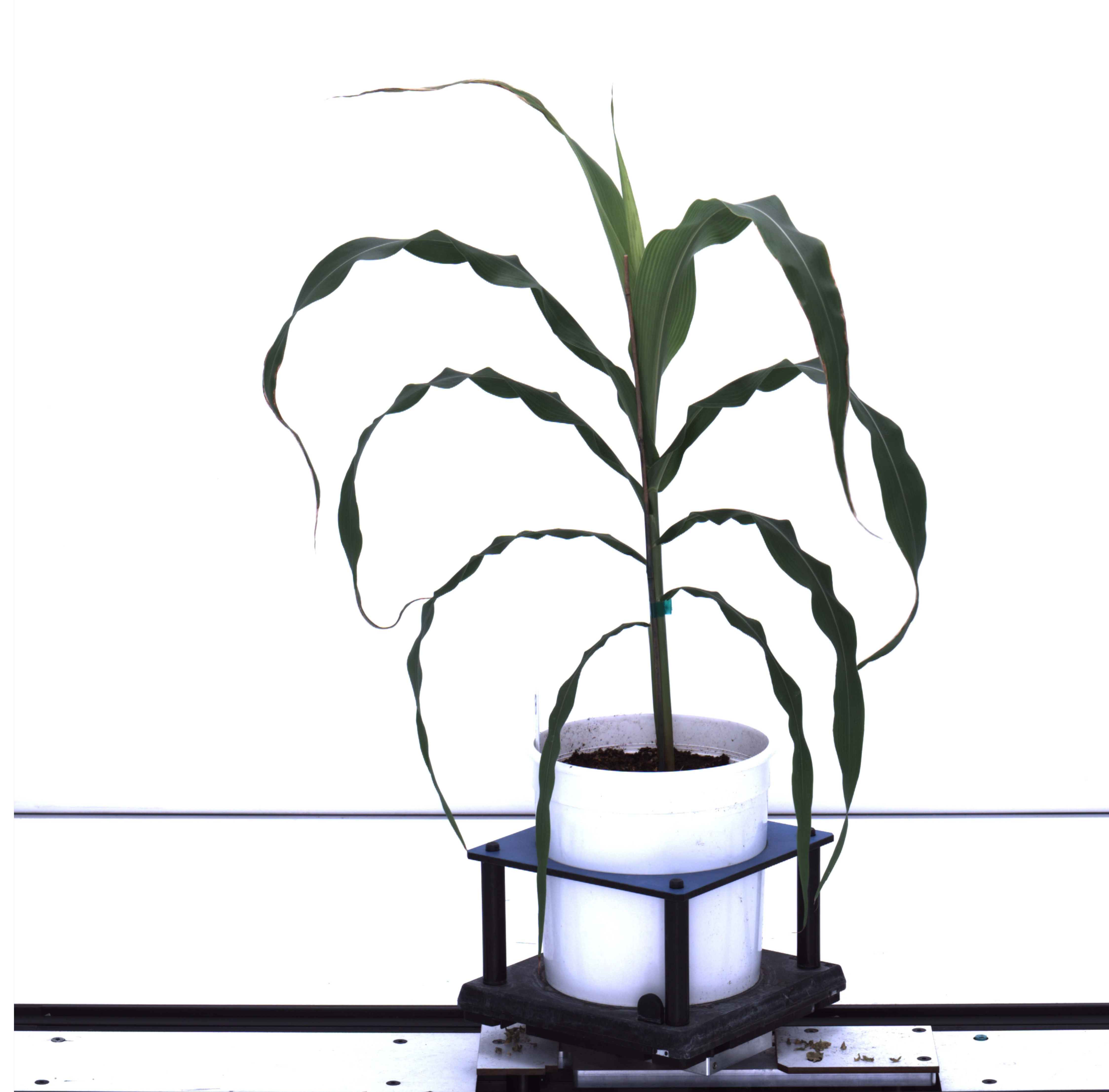

Supplement: Supplemental Information 1 — (A) BTx623 (B) AS 4601 Pawaga. [file peerj-09-12628-s001.pdf]

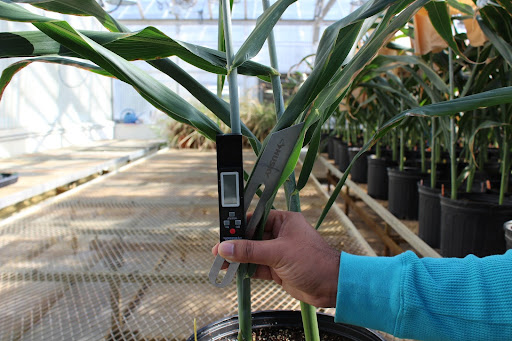

Supplement: Supplemental Information 2 — An electronic protractor (Husky Sliding Digital T-Bevel/Angle Finder) is used to measure the interior angle between the sorghum stem and the midrib of the target sorghum leaf. [file peerj-09-12628-s002.png]

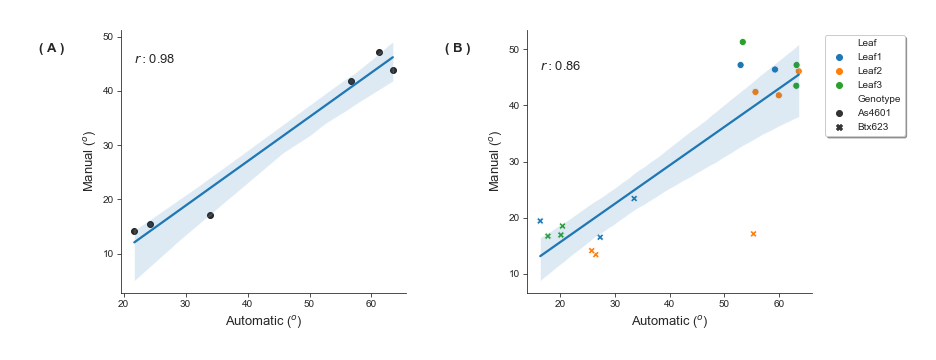

Supplement: Supplemental Information 3 — (A) Pearson r correlations between the median leaf angle of leaves 1 to 4 derived from manual measurements of six sorghum plants representing phenotypic extremes for leaf angle in sorghum versus the median leaf angle of the same plants derived from automatic measurements of the 3D reconstructed plants. (B) Pearson r correlations between manual measurements of the individual leaves 1 to 3 of six sorghum plants representing phenotypic extremes of leaf angle in sorghum versus automatic measurements of the same plants derived from 3D reconstructions. [file peerj-09-12628-s003.png]

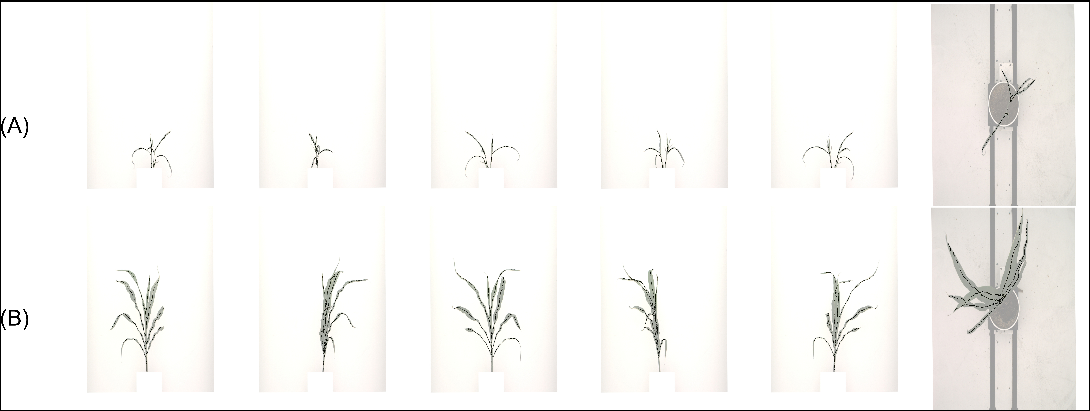

Supplement: Supplemental Information 4 — (A) Leaf angles were large due to biological errors as a result of extremely poor growth and health under greenhouse conditions. (B) Leaf angles for plant was inconsistent with visual validation. [file peerj-09-12628-s004.png]

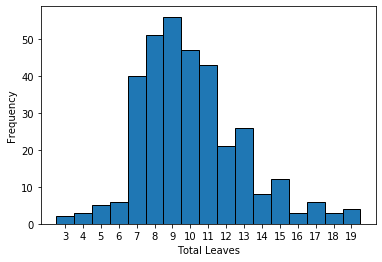

Supplement: Supplemental Information 5 [file peerj-09-12628-s005.png]

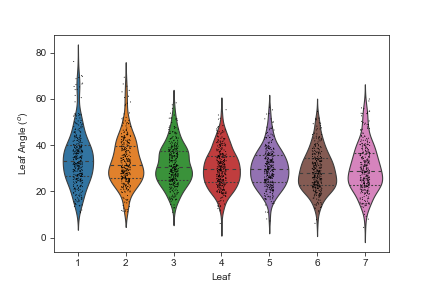

Supplement: Supplemental Information 6 [file peerj-09-12628-s006.png]

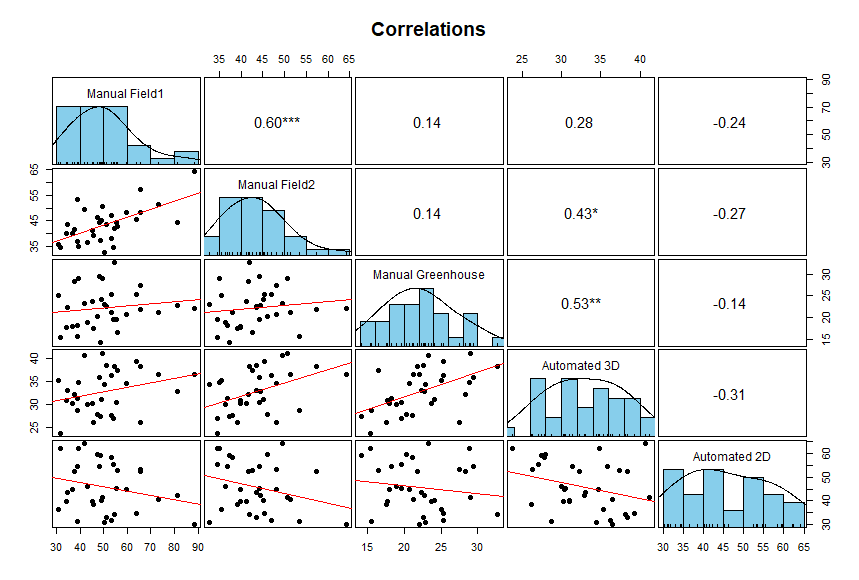

Supplement: Supplemental Information 7 — Manual Field 1, Manual Field 2, Automated 3D and Automated 2D refers to the Iowa 2012 manual field data, the Nebraska 2020 manual field data, the Nebraska 2021 manual green house data, the Nebraska 2018 greenhouse automatically derived 3D data and the Nebraska 2018 greenhouse automatically derived 2D data. Stronger correlations were observed between datasets grown under the same condition and maturity stage (Manual Field1 vs Manual Field2 and Manual greenhouse vs Automated 3D). Negative and non significant correlations observed between comparisons of automatically derived 2D measurements and all other datasets. [file peerj-09-12628-s007.png]

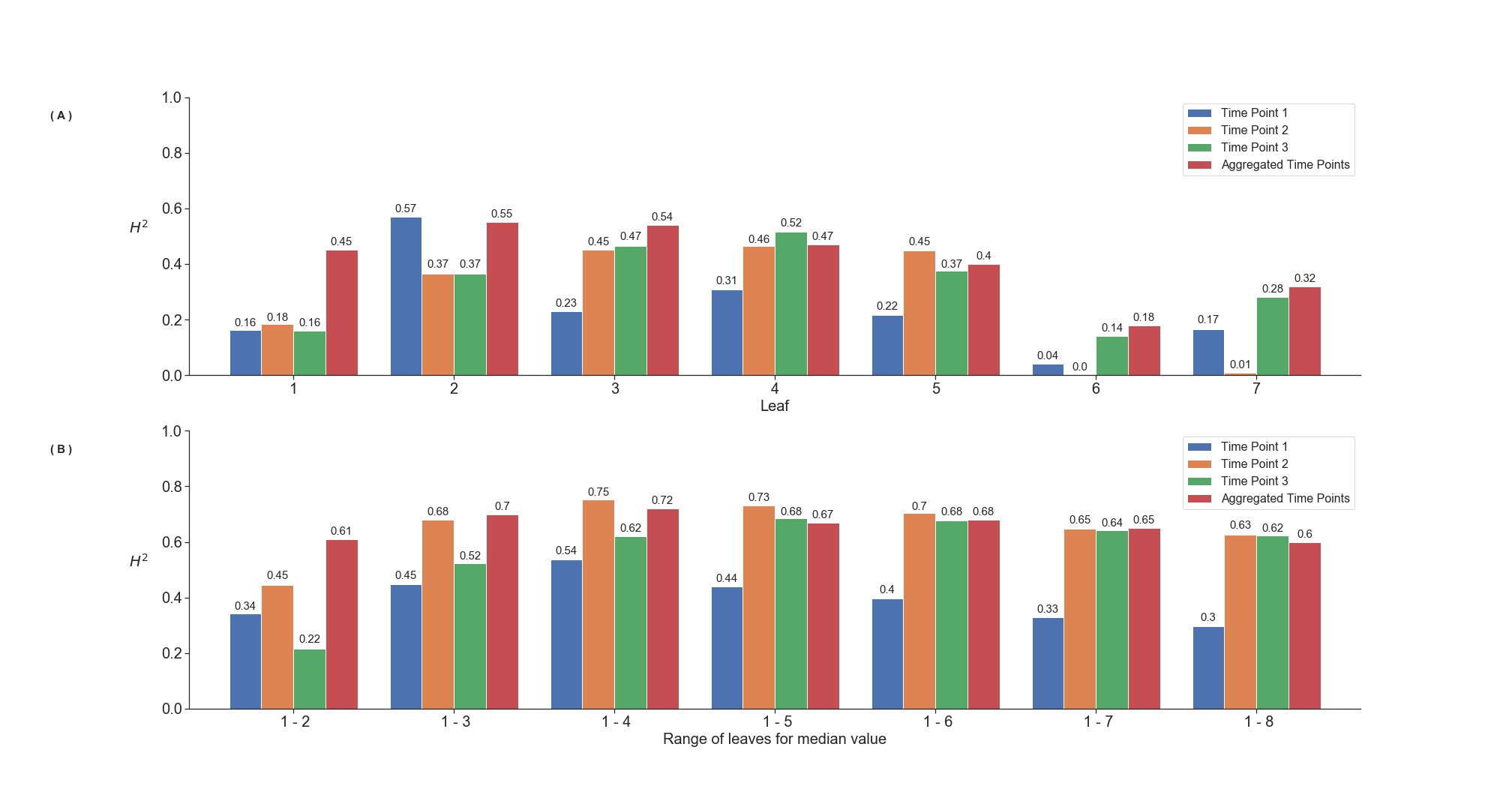

Supplement: Supplemental Information 8 — (A) Heritability calculated for each individual leaf for each of the three time points as well as after aggregation of time points. (B) Heritability calculated using the median value of different leaf combinations for three different time points and after aggregation (The median angle of the three values of a single leaf, was assigned for that specific leaf and plant). [file peerj-09-12628-s008.png]

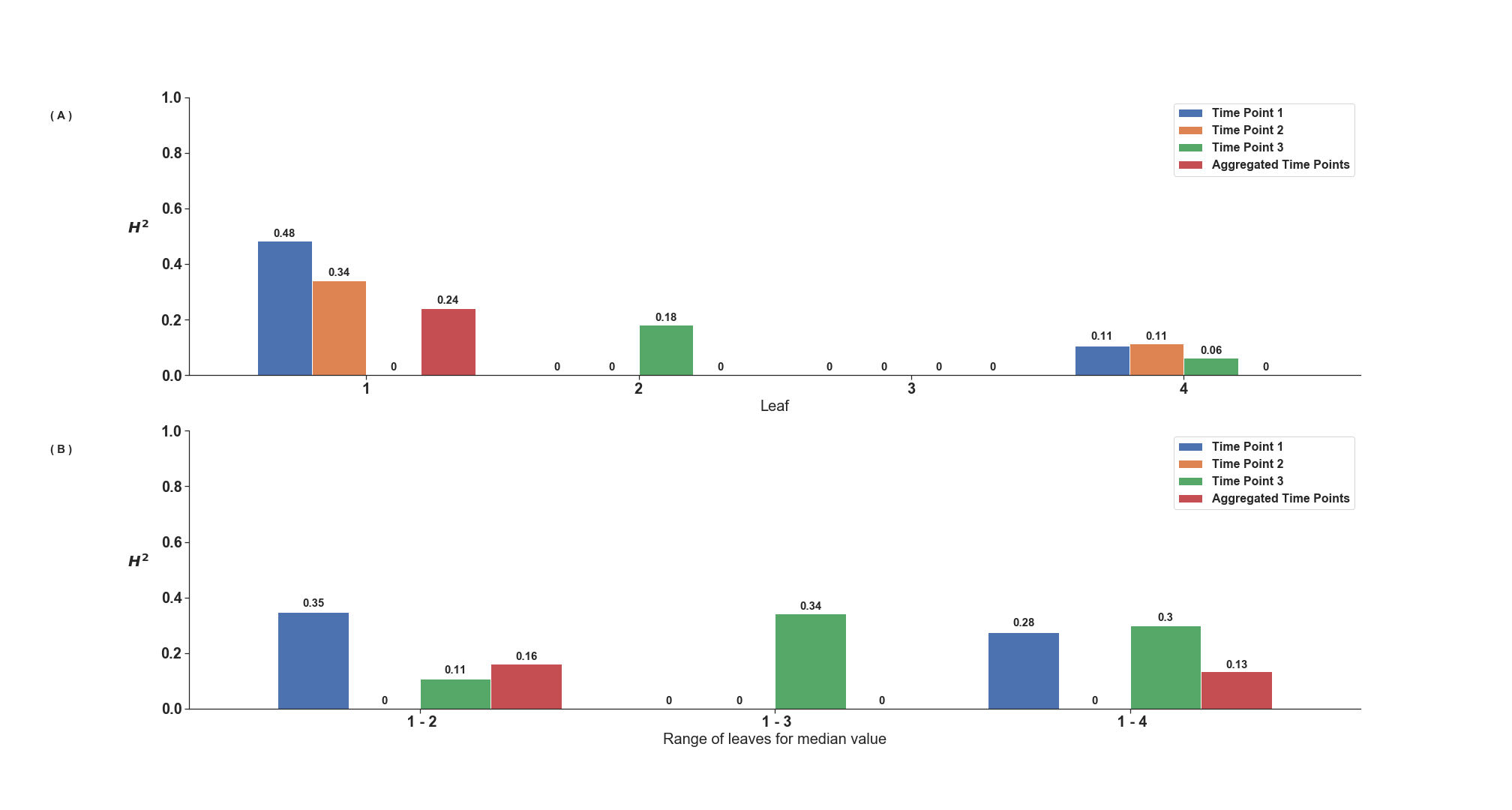

Supplement: Supplemental Information 9 — (A) Heritability calculated for each individual leaf for each of the three time points as well as after aggregation of time points. (B) Heritability calculated using the median value of different leaf combinations for three different time points and after aggregation (The median angle of the three values of a single leaf, was assigned for that specific leaf and plant). [file peerj-09-12628-s009.png]

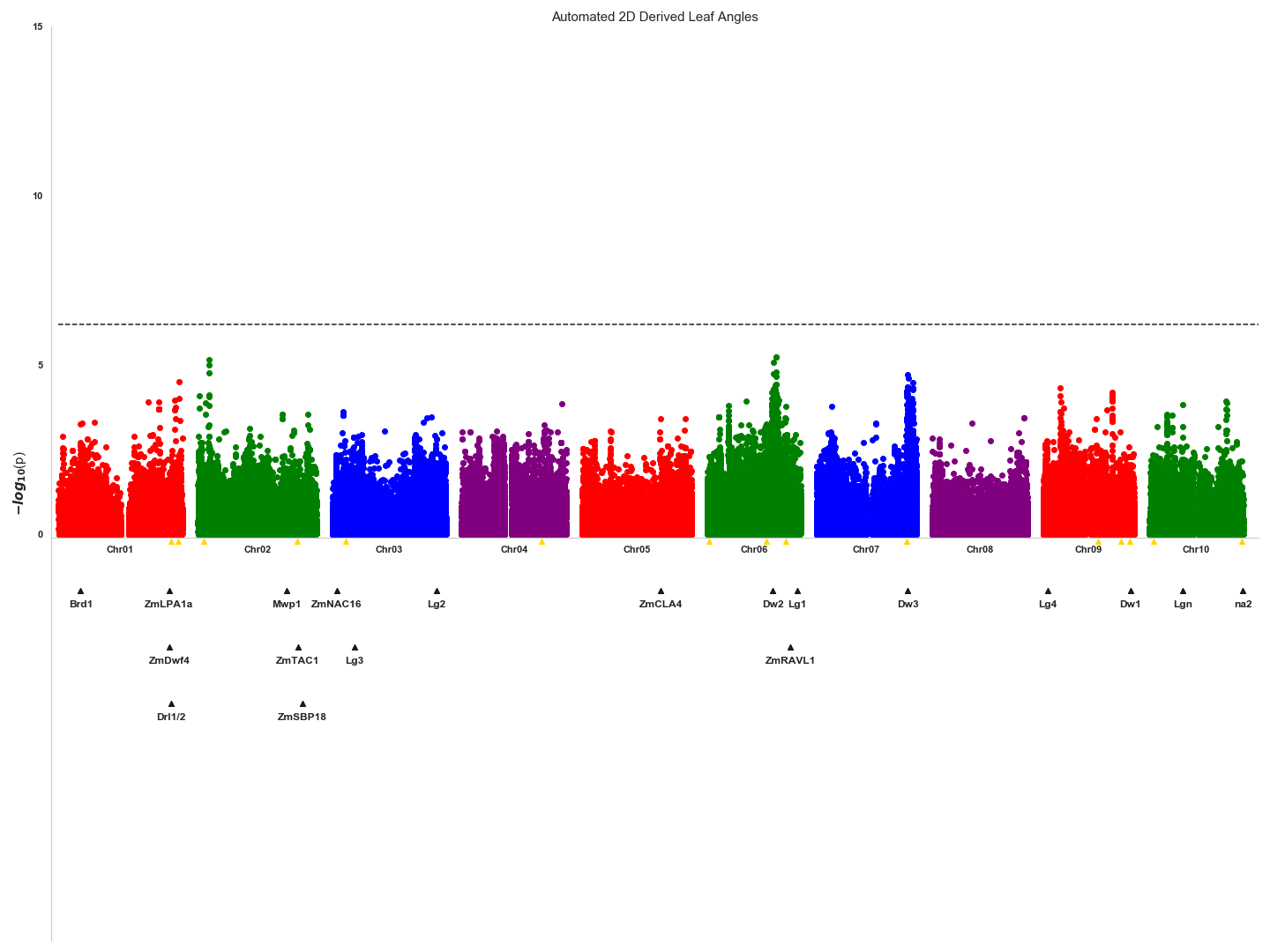

Supplement: Supplemental Information 10 — A Mixed linear model based approach implemented in GEMMA [39] was used to identify significant associations of SNP markers with variation in leaf angle aggregated across all three time points. Each point indicates the physical position and statistical significance of an individual marker. Dashed black lines indicate a genome wide threshold for statistical significance of a 6.39 × 10 −7 resulting from a bonferroni correction using an effective SNP number of 78,251 (See Methods). Yellow triangles indicate the locations of significant QTL for leaf erectness detected in an analysis of a sorghum NAM population (Olatoye, Hu & Morris, 2020. Black triangles indicate locations of a set of cloned sorghum genes or the locations of the syntenic ortholog in sorghum of maize genes which are known to influence variation in leaf angle in maize and are located near significantly trait associated SNPs in this study. [file peerj-09-12628-s010.png]

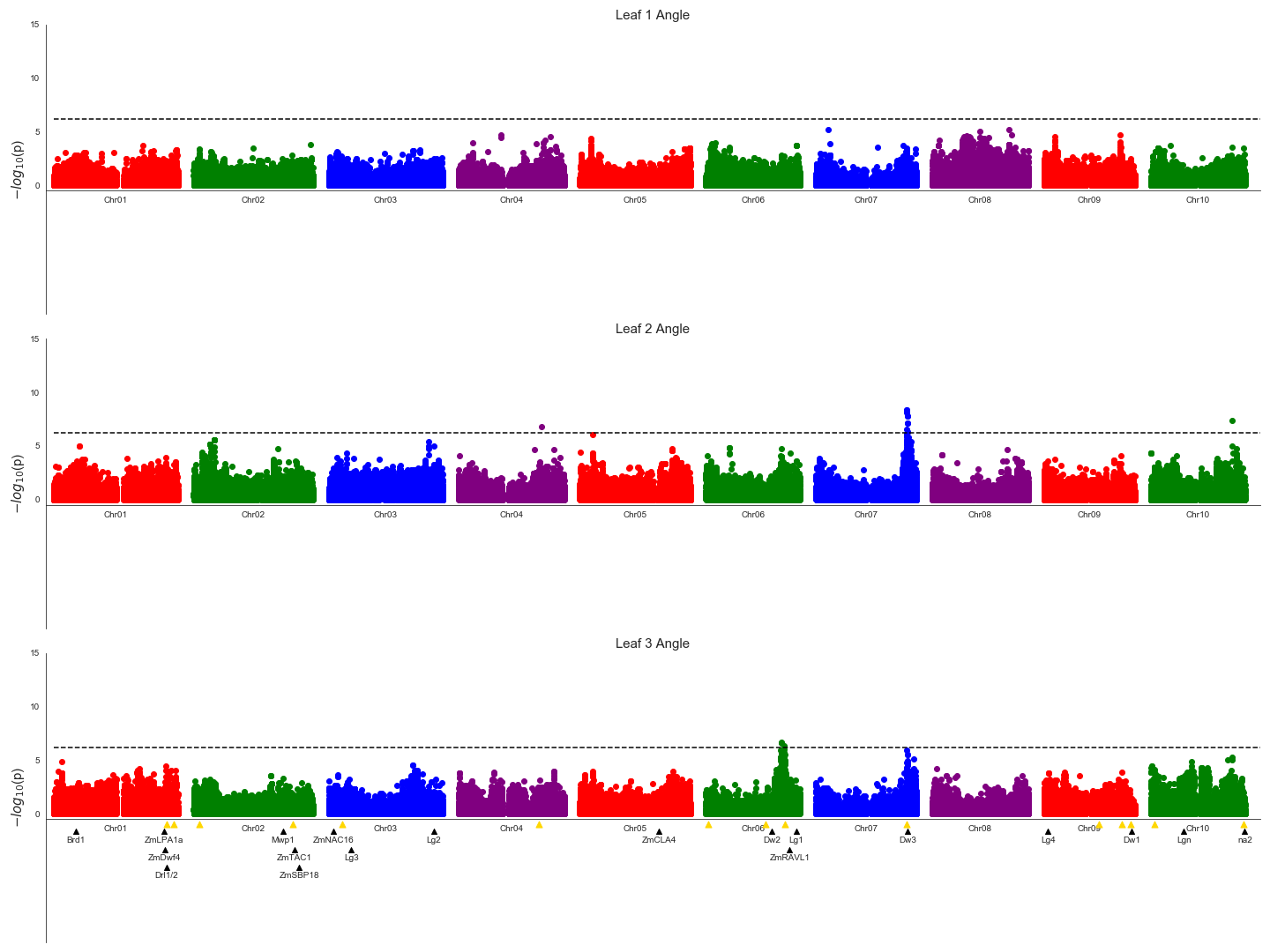

Supplement: Supplemental Information 11 — A Mixed linear model based approach implemented in GEMMA [39] was used to identify significant associations of SNP markers with variation in leaf angle aggregated across all three time points. Each point indicates the physical position and statistical significance of an individual marker. Dashed black lines indicate a genome wide threshold for statistical significance of a 6.39 × 10 −7 resulting from a bonferroni correction using an effective SNP number of 78,251 (See Methods). Yellow triangles indicate the locations of significant QTL for leaf erectness detected in an analysis of a sorghum NAM population (Olatoye, Hu & Morris, 2020. Black triangles indicate locations of a set of cloned sorghum genes or the locations of the syntenic ortholog in sorghum of maize genes which are known to influence variation in leaf angle in maize and are located near significantly trait associated SNPs in this study. [file peerj-09-12628-s011.png]
